# Developing Clinically Relevant Cut‐Off Values for Trunk Muscle Size in Relation to Lower Limb Injuries in AFL Players

**DOI:** 10.1155/tsm2/5549825

**Published:** 2026-09-21

**Authors:** Clint Frazer, M. Dilani Mendis, Peter Blanch, Brittany Grantham, Julie A. Hides

**Affiliations:** ^1^ Greater Western Sydney Australian Football Club, 1 Olympic Blvd Sydney Olympic Park, Sydney 2127, Australia; ^2^ School of Allied Health, Sport and Social Work, Griffith University, Nathan Campus Nathan, Brisbane 4111, Queensland, Australia, health.qld.gov.au; ^3^ Melbourne Football Club, AAMI Park, East Melbourne 8002, Victoria, Australia, health.qld.gov.au

**Keywords:** Australian Football, injury, multifidus, prevention, sport

## Abstract

**Objective:**

To investigate the relationship between trunk muscle morphology and lower limb injury risk in elite Australian Football League (AFL) players and to explore clinically interpretable cut‐off values for injury risk stratification.

**Design:**

Prospective cohort study.

**Method:**

Participants included 43 athletes from one professional AFL team (mean age 23.9 years, height 190 cm, mass 90.2 kg). Preseason ultrasound imaging was used to assess trunk muscle size and function, including cross‐sectional area (CSA) of the lumbar multifidus and quadratus lumborum (QL) and thickness of the transversus abdominis (TrA) and internal oblique (IO). Players were monitored over a full playing season for lower limb injuries resulting in games missed. Group comparisons and unadjusted logistic regression analyses were performed. Receiver operating characteristic (ROC) analyses were used to explore potential cut‐off values.

**Results:**

Smaller multifidus CSA (L_5_ average < 8.17 cm^2^; OR 10.0 [2.2–45.2]; *p* < 0.01) and larger QL CSA (average > 8.44 cm^2^; OR 7.3 [1.8–29.8]; *p* < 0.01) were associated with increased odds of lower limb injury. Trunk muscle thickness and contraction measures were not associated with lower limb or soft tissue injuries (all *p* > 0.05), with small and inconsistent effect sizes.

**Conclusion:**

Trunk muscle morphology is associated with lower limb injury risk in elite AFL players. These findings provide preliminary, clinically interpretable cut‐off values that may assist in identifying athletes at increased risk; however, these thresholds require validation in larger cohorts. Trunk muscle assessment may support injury‐risk screening and guide targeted physiotherapy interventions in applied high‐performance settings.

## 1. Introduction

Injuries in sport result in substantial financial and performance costs. In elite competitions such as the Australian Football League (AFL), injury burden has been linked to both financial loss and reduced team success, with teams finishing lower on the ladder with increasing injury rates [1–3]. As a result, identifying modifiable injury risk factors remains a key priority in sports medicine [4].

Lower limb injury risk factors can be classified as intrinsic or extrinsic, as well as modifiable or nonmodifiable. Modifiable intrinsic factors such as eccentric hamstring strength, muscle architecture and training workload have been widely investigated [5–7]. More recently, trunk muscle morphology and function have been explored as potential contributors to lower limb injury risk, based on the theory that effective trunk control facilitates efficient force transfer through the kinetic chain [8–11].

Trunk muscle size, particularly the cross‐sectional area (CSA) of the multifidus and quadratus lumborum (QL), has been identified as a potential risk factor for lower limb injuries in AFL players. Smaller multifidus CSA has consistently been associated with increased injury risk, with studies reporting increased odds of injury with decreasing muscle size [11, 12]. In contrast, larger QL CSA has also been associated with injury risk in both AFL and rugby league athletes [12–14], although conflicting findings exist [15]. These inconsistencies may reflect sport‐specific demands and differences in athlete characteristics, highlighting the importance of establishing sport‐specific clinical cut‐off values.

Muscle function has also been investigated as a risk factor, commonly assessed using ultrasound measures of thickness and contraction. Findings have been mixed, with some studies demonstrating associations between impaired trunk muscle function and injury [16–18], while others have reported no relationship [14]. These discrepancies suggest that muscle size and muscle function may represent different aspects of neuromuscular performance.

To date, no study has comprehensively examined both trunk muscle size across multiple lumbar levels and measures of muscle contraction in relation to lower‐limb injuries in AFL athletes. The authors hypothesise that smaller multifidus muscle CSA and larger QL muscle CSA will be associated with increased lower limb injury risk and that reduced trunk muscle thickness and contraction measures will also increase injury risk. This study aims to identify clinically relevant cut‐off values that may assist in identifying AFL players who warrant further assessment of trunk muscle characteristics.

Despite growing interest in trunk muscle morphology and function, there remains limited evidence to guide how these measures can be applied in practice to identify athletes at risk of injury and inform targeted interventions. A clearer understanding of the relative importance of muscle size and function may assist clinicians, coaches and support staff in prioritising screening strategies and individualising training or rehabilitation programmes.

The authors hypothesise that smaller multifidus muscle CSA and larger QL CSA will be associated with lower limb injuries. Additionally, reduced trunk muscle thickness and contraction measures are expected to be associated with an increased risk of injury. Findings from this study may help inform the identification of athletes who warrant further assessment of trunk muscle characteristics and support the development of more targeted intervention strategies.

## 2. Method

### 2.1. Participants

**FIGURE 1:**
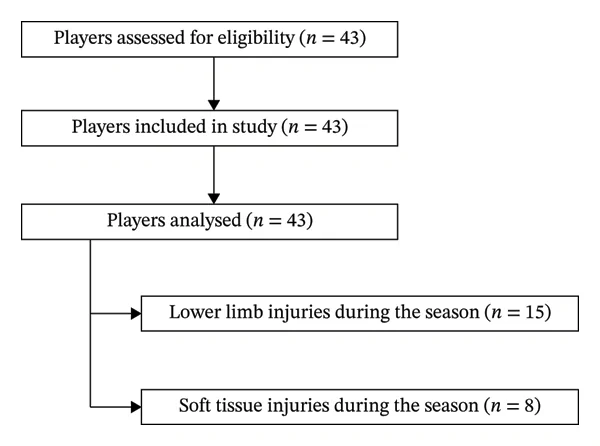
Flow diagram of participant inclusion, analysis and injury outcomes.

Forty‐three players from one professional AFL club were invited to participate in the study, and no players declined participation. Participant recruitment, inclusion in the analysis and injury outcomes are summarised in Figure 1. Participants were assessed in the preseason, and injury data were collected across the subsequent playing season. Written informed consent was gained from all players included in the study. Players were excluded if they had previously undergone lumbar spine surgery, while players with low back pain and other past surgical and injury histories were included in the study. This study was approved by the Griffith University Human Research Ethics Committee’s ‘Multi‐Disciplinary Approach to Modelling and Prevention of Sports Injuries’ (2017/896). Funding provided by Griffith University Health Group & Brisbane Lions FC: Fostering Research Partnerships Scheme to support research assistants for data collection.

### 2.2. Ultrasound Imaging Protocols

#### 2.2.1. Multifidus Muscle (CSA)

The protocols for ultrasound imaging of the multifidus muscles in cross‐section have been published in detail [10]. All imaging assessments were conducted under standardised conditions during the preseason period. All ultrasound measurements were performed by a single experienced assessor who has previously established intra‐ and inter‐rater reliability for these measurement protocols using identical procedures. While imaging was not standardised by time of day or fatigue state, all participants were assessed under similar preseason conditions, minimising variability. In the present study, all imaging assessments were conducted during the preseason, prior to injury occurrence and image analysis was performed without knowledge of subsequent injury outcomes, thereby reducing the risk of measurement bias.

**FIGURE 2:**
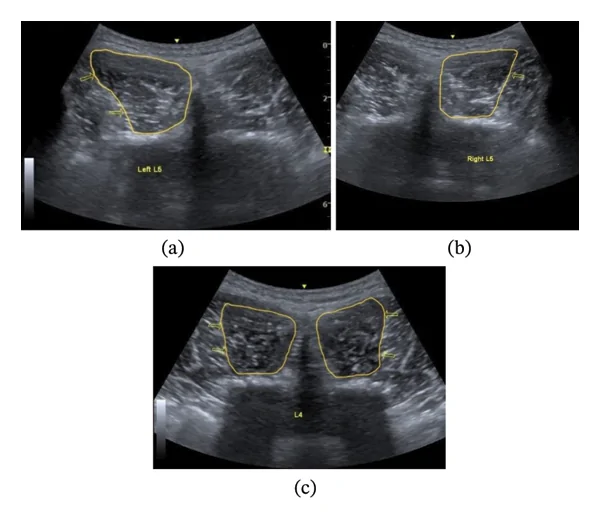
Ultrasound images of (a) left L_5_ multifidus cross‐sectional area, (b) right L_5_ multifidus cross‐sectional area and (c) bilateral L_4_ multifidus cross‐sectional area.

Players were positioned in prone lying with a pillow placed under the abdomen to reduce the lumbar lordosis. The spinal level was identified by palpating and marking the lumbar spinous process from L_2_ to L_5_ vertebral levels. The multifidus muscles were imaged in the transverse plane, bilaterally from L_2_ to L_5_. Where the field of view was limited, bilateral images, left and right sides, were imaged separately. Ultrasound imaging was conducted using a LOGIQ e apparatus with a 5‐MHz curvilinear transducer (GE Healthcare, Wuxi, China). All images were captured by researchers with proven reliability in the measure. Ultrasound images were stored and measured offline using OsiriX medical imaging software (Geneva, Switzerland). The CSA of the multifidus was measured by manually tracing the outline of the muscle. The landmarks used to identify the borders of the MF muscle were the shadow of the tip of the spinous process (medial border), the echogenic lamina (inferiorly), the thoracolumbar fascia (superiorly) and the fascia between the MF muscle and the lumbar longissimus muscle (laterally). The researcher was blinded to the identity of each participant and had previously demonstrated reliability in conducting the CSA measurements of the multifidus muscles (ICC mean L_2_–L_5_ = 0.94) (Figure 2).

#### 2.2.2. QL Muscle (CSA)

**FIGURE 3:**
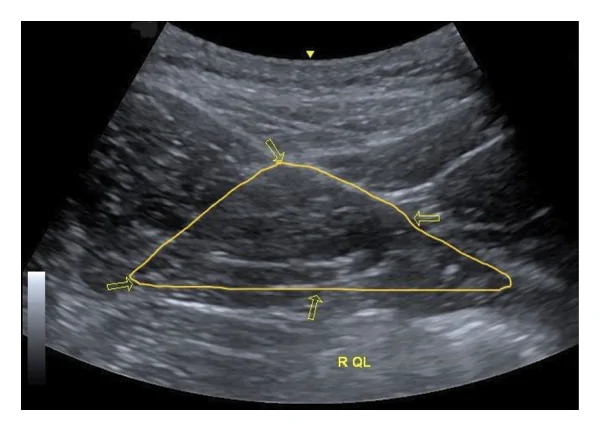
Ultrasound image of the right quadratus lumborum cross‐sectional area.

The lumbar spinous processes were palpated and marked with a pen prior to imaging. The QL muscle was imaged by moving the transducer laterally in line with the L_3–4_ vertebral interspace on each side. The border of the QL muscle was defined by the lateral tip of the transverse process and lumbar erector spinae medially, the fascia of the lateral abdominal wall laterally and the border of the psoas major deep to the QL muscle (Figure 3). Measures of the QL muscle showed high reliability (ICC = 0.99) and were very consistent across two time points (*F* = 0.26, *p* = 0.61) [13].

#### 2.2.3. Transversus Abdominis (TrA) and Internal Oblique (IO) Muscles (Thickness Measures)

**FIGURE 4:**
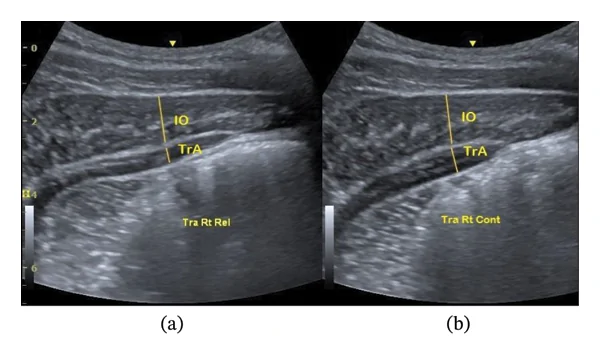
Ultrasound images of (a) relaxed internal oblique and transversus abdominis muscle thickness and (b) contracted internal oblique and transversus abdominis muscle thickness.

Ultrasound imaging of the TrA and IO muscles was conducted in the supine lying position. The transducer was placed perpendicular to the fascia covering the anterolateral abdominal muscles and positioned midway between the inferior angle of the ribcage and the iliac crest. To standardise the position of the transducer, the anterior fascial insertion of the TrA muscle was positioned approximately 2 cm from the medial edge of the ultrasound image. Images were captured at rest and on contraction, with the instruction ‘relax the abdominal wall, take a relaxed breath in, breathe out, hold the breath out and then slowly and gently draw in the abdominal wall without moving the spine’. Measures of muscle thickness were conducted on stored images using OsiriX (Figure 4).

#### 2.2.4. Multifidus Muscle (Thickness Measurements)

**FIGURE 5:**
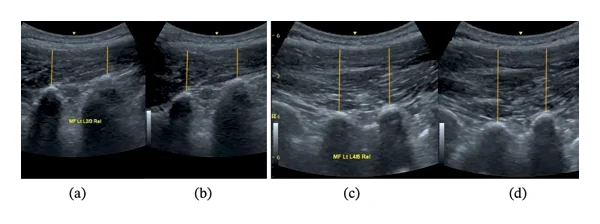
Ultrasound images of (a) L_2_–L_3_ relaxed multifidus muscle thickness, (b) L_2_–L_3_ contracted multifidus muscle thickness, (c) L_4_–L_5_ relaxed multifidus muscle thickness and (d) L_4_–L_5_ contracted multifidus muscle thickness.

Imaging of the multifidus was performed in a parasagittal section. This allowed visualisation of L_2_–L_5_ zygapophyseal joints, muscle bulk and thoracolumbar fascia. Images were captured at rest and on contraction. For the voluntary isometric contraction of the multifidus muscle, the examiner placed their fingers over the muscle and asked the player to ‘relax the trunk muscles, then take a relaxed breath in and out, hold the breath out and then try to contract or swell the multifidus muscle into the examiner’s fingers’. The multifidus muscle was then imaged at each vertebral level from L_2_–L_5_ in the resting and contracted states. Linear measurements of stored images were conducted using OsiriX, from the zygapophyseal joints to the superior border of the multifidus (thoracolumbar fascia) (Figure 5).

#### 2.2.5. Injury Data

Injury data were collected by the medical staff of the AFL club involved. Injuries were defined as those resulting in missed matches. Injuries were defined as those resulting in missed matches, reflecting a pragmatic and clinically relevant definition commonly used in AFL injury surveillance. Both contact and noncontact injuries were included. Players who sustained multiple or recurrent injuries were classified as a single injured case for analysis. Injuries that required training modification but did not result in missed games were not included. Four players were managing injuries at the time of the preseason assessment and were not reimaged upon return to full training. Previous injury history was recorded; however, it was not included in the analysis, and only a history of spinal surgery was considered an exclusion criterion. Player exposure to training and matches was not quantified, representing a potential source of confounding, as differences in workload may influence both trunk muscle characteristics and injury risk.

### 2.3. Statistical Analysis

SPSS Version 30 (IBM) was used for all analyses. No a priori sample size calculation was performed, as the study was conducted within a fixed cohort of professional AFL players from a single team. Data were examined for outliers and normal distribution using summary statistics, histograms, normality plots and the Kolmogorov–Smirnov test. Normality was assessed using visual inspection of histograms and Q–Q plots in combination with the Kolmogorov–Smirnov test. Given the relatively small sample size, visual inspection was prioritised when interpreting distributional assumptions. Homogeneity of variance was assessed using Levene’s test. Where assumptions were met, independent samples *t*‐tests were used. One player who sustained a soft‐tissue injury had missing data for TrA and IO thickness measurements due to a data storage error. The data were deleted for analysis for these two variables, reducing the number from 43 to 42 players. This represents 2% of the total observations. The measures tested as risk factors included age, height, mass, muscle CSA and contraction measures. Both left and right muscle CSAs for the multifidus and QL muscles and thickness measures (for the TrA, IO and multifidus) were analysed as well as averaged for each measurement. All factors were assessed against ‘lower limb’ and ‘soft‐tissue injuries’ in the playing season. A two‐sample *t*‐test was used to compare the ‘lower limb injury’ and ‘no lower limb injury’ groups and ‘soft tissue’ and ‘no soft tissue injury’ groups for all continuous variables (age, mass, height, muscle CSA and muscle function measures). Cohen’s d was used to present the effect size of each variable with 95% confidence intervals. If a significant difference between means was found, receiver operating characteristic (ROC) curves were used to determine an optimal cut point for each continuous variable in predicting the injury outcome variable. The optimal cut point was obtained as the point where the true‐positive rate (sensitivity) was maximised and the false‐positive rate (1‐specificity) was minimised, that is, the point closest to the top of the *y*‐axis. ROC curve analysis was conducted to identify a clinical cut‐off value for both lower limb injuries and soft tissue injuries. Area under the curve and 95% confidence intervals were calculated. The cut points were then used to convert the continuous variables to a binary form. Cross‐tabulations and chi‐square tests were used to calculate sensitivity and specificity for each variable. The unadjusted odds ratios for predicting lower limb and soft tissue injuries were calculated.

## 3. Results

Forty‐three male AFL players had their trunk muscle size and function measured at the start of the season. Mean participant age, height and mass were 23.9 ± 3.8 years, 190.0 ± 7.4 cm and 90.2 ± 7.9 kg. Participants were followed prospectively across the 2018 AFL playing season (24 weeks), with all injuries recorded during this period. Fifteen players suffered a lower limb injury (eight muscle soft tissue, two ankle, two foot, two leg and one knee), while eight of these injuries involved soft tissue injuries (five hamstring, one groin and two calf injuries).

### 3.1. Injuries and Trunk Muscle Size

Players who missed games due to lower‐limb injuries during the playing season had smaller L_3_–L_5_ multifidus CSA at the start of the season than players with no injuries (Table 1). While L_3_ and L_4_ multifidus CSA were significantly different between the groups, the largest effect sizes were seen at the L_5_ vertebral level (Table 1). In contrast, a larger QL CSA was observed in players with a lower‐limb injury during the season (Table 1).

**TABLE 1:** Multifidus cross‐sectional area of players who received a lower limb injury in season.

Variable	No lower limb injury Mean (SD)				Lower limb injury Mean (SD)				Effect size (CI 95%)			*p* value		
	*N*	Left	Right	Average	*N*	Left	Right	Average	Left	Right	Average	Left	Right	Average
MF L_2_ CSA	28	2.72 (0.62)	2.74 (0.65)	2.73 (0.63)	15	2.33 (0.48)	2.41 (0.48)	2.37 (0.48)	0.69 (0.04–1.30)	0.56 (−0.08–1.19)	0.63 (−0.02–1.26)	**0.03**	0.06	**0.04**
MF L_3_ CSA	28	3.95 (0.90)	3.89 (0.89)	3.92 (0.90)	15	3.24 (0.75)	3.20 (0.77)	3.22 (0.75)	0.82 (0.17–1.47)	0.81 (0.16–1.46)	0.82 (0.16–1.47)	**0.01**	**0.01**	**0.01**
MF L_4_ CSA	28	8.07 (2.31)	8.20 (2.42)	8.14 (2.34)	15	6.63 (0.80)	6.66 (1.15)	6.65 (0.94)	0.75 (0.09–1.39)	0.74 (0.09–1.37)	0.75 (0.10–1.40)	**< 0.01**	**< 0.01**	**< 0.01**
MF L_5_ CSA	28	9.36 (1.86)	9.48 (2.00)	9.42 (1.90)	15	7.60 (1.09)	7.82 (1.10)	7.71 (1.04)	1.07 (0.40–1.74)	0.95 (0.28–1.60)	1.03 (0.36–1.69)	**< 0.001**	**< 0.01**	**< 0.001**
QL CSA	28	7.72 (1.60)	7.56 (1.50)	7.64 (1.40)	15	8.97 (0.91)	8.86 (1.59)	8.91 (1.09)	−0.89 (−1.54–−0.23)	−0.85 (−1.45–−0.19)	−0.98 (−1.63–−0.31)	**< 0.01**	**0.02**	**< 0.01**

*Note:* Bold values indicate statistically significant differences between groups (*p* ≤ 0.05). MF, multifidus.

Abbreviations: CI, confidence interval; CSA, cross‐sectional area; QL, quadratus lumborum; SD, standard deviation.

Further analysis of the subset of soft tissue injuries found there was a similar association between a smaller multifidus CSA and soft tissue injuries (Table 2). Players who sustained a soft tissue injury demonstrated smaller multifidus CSA at L_2_, L_4_ and L_5_ compared with those who did not sustain injury, with corresponding *p* values indicating statistical significance. There was evidence of a difference; however, the confidence intervals were wide and, in some cases, approached or crossed the null value, indicating uncertainty in the estimates. The QL CSA was not significant for soft tissue injuries in this cohort (Table 2).

**TABLE 2:** Multifidus cross‐sectional area of players who received a soft tissue injury in season.

Variable	No soft tissue injury Mean (SD)				Soft tissue injury Mean (SD)				Effect size (CI 95%)			*p* value		
	*N*	Left	Right	Average	*N*	Left	Right	Average	Left	Right	Average	Left	Right	Average
MF L_2_ CSA	35	2.68 (0.60)	2.70 (0.62)	2.69 (0.61)	8	2.20 (0.44)	2.30 (0.44)	2.24 (0.43)	0.84 (0.05–1.63)	0.68 (−0.11–1.45)	0.76 (−0.03–1.54)	**0.02**	**0.05**	**0.03**
MF L_3_ CSA	35	3.80 (0.91)	3.74 (0.91)	3.77 (0.91)	8	3.29 (0.80)	3.26 (0.85)	3.28 (0.82)	0.57 (−0.22–1.34)	0.53 (−0.25–1.30)	0.55 (−0.23–1.33)	0.14	0.18	0.16
MF L_4_ CSA	35	7.81 (2.14)	7.92 (2.29)	7.86 (2.19)	8	6.53 (0.96)	6.54 (1.15)	6.53 (1.03)	0.64 (−0.14–1.42)	0.65 (−0.14–1.43)	0.65 (−0.13–1.43)	**0.02**	**0.02**	**0.02**
MF L_5_ CSA	35	8.97 (1.89)	9.12 (2.01)	9.05 (1.92)	8	7.77 (1.17)	7.94 (0.88)	7.86 (0.97)	0.67 (−0.12–1.45)	0.63 (−0.15–1.41)	0.66 (−0.12–1.44)	**0.04**	**0.02**	**0.02**
QL CSA	35	8.00 (1.57)	7.85 (1.51)	7.93 (1.39)	8	8.83 (0.99)	8.75 (2.02)	8.79 (1.47)	−0.55 (−1.38–0.23)	−0.56 (−1.34–0.22)	−0.62 (−1.40–0.17)	0.08	0.26	0.16

*Note:* The bold values are those with a *p* value < 0.05. MF, multifidus.

Abbreviations: CI, confidence interval; CSA, cross‐sectional area; QL, quadratus lumborum; SD, standard deviation.

Analysis of ROC curves for variables that reached significance for a relationship with lower limb and soft tissue injury is presented in Tables 3 and 4. The lower‐limb injury AUC for multifidus CSA reached significance at the L_3_–L_5_ levels. The highest sensitivity and specificity were also consistently found at the L_5_ multifidus level (Table 3). The QL AUC also reached significance, with a high sensitivity and specificity indicating a relationship to lower limb injuries (Table 3). Multifidus CSA at L_2_, L_4_ and L_5_ had a significant AUC for soft tissue injuries. Like lower limb injuries, the more significant findings were at the L_5_ multifidus level (Table 4).

**TABLE 3:** ROC curves optimal cut values for variables associated with lower limb season injuries.

Variable	AUC	95% confidence interval	*p* value	Optimal risk cut‐point	Sensitivity (%)	Specificity (%)
MF L_2_ CSA left (cm^2^)	0.67	(0.50, 0.83)	0.05	2.67	50	80
MF L_2_ CSA right (cm^2^)	0.64	(0.47, 0.83)	0.11	2.60	57	67
MF L_2_ CSA average (cm^2^)	0.65	(0.48, 0.82)	0.08	2.54	57	67
MF L_3_ CSA left (cm^2^)	0.70	(0.55, 0.86)	0.01	3.63	64	73
MF L_3_ CSA right (cm^2^)	0.70	(0.54, 0.86)	0.01	3.60	64	73
MF L_3_ CSA average (cm^2^)	0.70	(0.54, 0.86)	0.01	3.61	64	73
MF L_4_ CSA left (cm^2^)	0.70	(0.55, 0.86)	< 0.01	7.39	54	93
MF L_4_ CSA right (cm^2^)	0.71	(0.55, 0.86)	< 0.01	6.51	75	53
MF L_4_ CSA average (cm^2^)	0.71	(0.55, 0.86)	< 0.01	7.56	54	87
MF L_5_ CSA left (cm^2^)	0.72	(0.65, 0.92)	< 0.01	8.65	68	93
MF L_5_ CSA right (cm^2^)	0.71	(0.62, 0.90)	< 0.01	8.57	68	80
MF L_5_ CSA average (cm^2^)	0.72	(0.65, 0.92)	< 0.01	8.17	71	80
QL left CSA (cm^2^)	0.75	(0.60, 0.90)	< 0.01	8.44	73	71
QL right CSA (cm^2^)	0.71	(0.55, 0.87)	< 0.01	7.96	73	57
QL average CSA (cm^2^)	0.78	(0.63, 0.92)	< 0.01	8.44	73	79

*Note:* MF, multifidus, cm, centimetre.

Abbreviations: AUC, area under the curve; CSA, cross‐sectional area; QL, quadratus lumborum.

**TABLE 4:** ROC curves optimal cut values for variables associated with soft tissue season injuries.

Variable	AUC	95% confidence interval	*p* value	Optimal risk cut‐point	Sensitivity (%)	Specificity (%)
MF L_2_ CSA left (cm^2^)	0.73	(0.54, 0.92)	0.02	2.35	69	75
MF L_2_ CSA right (cm^2^)	0.70	(0.51, 0.89)	0.04	2.23	77	63
MF L_2_ CSA average (cm^2^)	0.72	(0.53, 0.91)	0.02	2.11	83	63
MF L_4_ CSA left (cm^2^)	0.69	(0.51, 0.88)	0.04	6.50	71	63
MF L_4_ CSA right (cm^2^)	0.70	(0.52, 0.89)	0.03	6.11	83	63
MF L_4_ CSA average (cm^2^)	0.70	(0.51, 0.88)	0.04	6.20	80	63
MF L_5_ CSA left (cm^2^)	0.72	(0.55, 0.89)	0.01	7.51	80	63
MF L_5_ CSA right (cm^2^)	0.71	(0.55, 0.87)	0.01	8.40	63	88
MF L_5_ CSA average (cm^2^)	0.72	(0.56, 0.89)	< 0.01	7.88	74	75

*Note:* MF, multifidus; cm, centimetre.

Abbreviations: AUC, area under the curve; CSA, cross‐sectional area; QL, quadratus lumborum.

The unadjusted odds ratios for injury risk factors associated with lower‐limb injuries are presented in Table 5. The odds ratio values shown in Table 5 indicate that a player with a smaller multifidus CSA (L_5_ average < 8.17 cm^2^ OR 10.0 (2.2–45.2); *p* < 0.01) would have a higher odds of suffering an in‐season lower‐limb injury. The reverse relationship was found for QL, where a larger CSA (QL average > 8.44 cm^2^ OR 7.3 (1.8–29.8); *p* < 0.01) increased the odds of an in‐season lower‐limb injury. The unadjusted odds ratios for the injury risk factors associated with soft tissue injuries are presented in Table 6. Only the results at the L_5_ multifidus level reached significance, indicating a higher chance of soft tissue injury with a smaller L_5_ multifidus muscle CSA (L_5_ average < 7.88 cm^2^ OR 8.3 (0.9–74.9); *p* = 0.03).

**TABLE 5:** Unadjusted odds ratios for multifidus and quadratus lumborum muscle cross‐sectional area for lower limb injuries.

Variable		Players ‘at risk’ with lower leg injury (%)	Players ‘nonrisk’ with lower limb injury (%)	Unadjusted odds ratio	*p* value
Multifidus L_2_ CSA	Left < 2.67 cm^2^	82.0	18.0	4.0 (0.9–17.3)	0.06
	Right < 2.60 cm^2^	45.5	23.8	2.70 (0.7–9.9)	0.14
	Average < 2.54 cm^2^	45.5	23.8	2.70 (0.7–9.9)	0.14

Multifidus L_3_ CSA	Left < 3.63 cm^2^	52.4	18.2	5.0 (1.2–19.7)	**0.02**
	Right < 3.60 cm^2^	52.4	18.2	5.0 (1.2–19.7)	**0.02**
	Average < 3.61 cm^2^	52.4	18.2	5.0 (1.2–19.7)	**0.02**

Multifidus L_4_ CSA	Left < 7.39 cm^2^	53.8	5.9	18.7 (2.1–162.3)	**0.01**
	Right < 6.51 cm^2^	53.3	25.0	3.4 (0.9–12.9)	0.06
	Average < 7.56 cm^2^	54.2	10.5	10.0 (1.9–53.4)	**0.03**

Multifidus L_5_ CSA	Left < 8.65 cm^2^	54.5	14.3	7.2 (1.6–31.7)	**< 0.01**
	Right < 8.57 cm^2^	57.1	13.6	8.4 (1.9–37.6)	**< 0.01**
	Average < 8.17 cm^2^	60.0	13.0	10.0 (2.2–45.2)	**< 0.01**

Quadratus lumborum CSA	Left > 8.44 cm^2^	57.9	16.7	6.9 (1.7–28.1)	**< 0.01**
	Right > 7.96 cm^2^	47.8	20.0	3.7 (0.9–14.4)	0.06
	Average > 8.44 cm^2^	62.5	18.5	7.3 (1.8–29.8)	**< 0.01**

*Note:* Bold values indicate statistically significant results (*p* ≤ 0.05). cm, centimetre.

Abbreviation: CSA, cross‐sectional area.

**TABLE 6:** Unadjusted odds ratios for multifidus muscle cross‐sectional area for soft tissue injuries.

Variable		Players ‘at risk’ with soft tissue injury (%)	Players ‘nonrisk’ with soft tissue injury (%)	Unadjusted odds ratio	*p* value
Multifidus L_2_ CSA	Left < 2.35 cm^2^	22.2	12.5	2.0 (0.4–11.4)	0.43
	Right < 2.23 cm^2^	27.3	9.5	3.6 (0.6–20.2)	0.14
	Average < 2.11 cm^2^	27.3	9.5	3.6 (0.6–20.2)	0.14

Multifidus L_4_ CSA	Left < 6.50 cm^2^	26.9	5.9	5.9 (0.7–53.1)	0.08
	Right < 6.11 cm^2^	27.8	12.0	2.8 (0.6–13.8)	0.19
	Average < 6.20 cm^2^	23.1	11.8	2.3 (0.4–12.8)	0.35

Multifidus L_5_ CSA	Left < 7.51 cm^2^	30.4	5.0	8.3 (0.9–74.9)	**0.03**
	Right < 8.18 cm^2^	33.3	4.5	10.5 (1.2–94.9)	**0.02**
	Average < 7.88 cm^2^	30.4	5.0	8.3 (0.9–74.9)	**0.03**

*Note:* Bold values indicate statistically significant results (*p* < 0.05).

### 3.2. Injuries and Trunk Muscle Function

Thickness of the multifidus, TrA and IO muscles at rest and when contracted was not associated with seasonal lower limb injuries (Table 7). Also, the percentage difference between the resting thickness and contracted thickness of these muscles was not associated with seasonal lower limb injuries. Soft tissue injuries also were not associated with muscle thickness measures of the multifidus, TrA and IO muscles (Table 8).

**TABLE 7:** Trunk muscle thickness and function of players who received a lower limb injury in the playing season.

Variable	No lower limb injury Mean (SD)			In‐season lower limb injury Mean (SD)				Effect size (CI 95%)		*p* value	
	*N*	Left	Right	*N*	Left	*N*	Right	Left	Right	Left	Right
MF L_2_ rest	28	2.62 (0.41)	2.70 (0.40)	15	3.18 (1.61)	15	2.69 (0.38)	−0.56 (−1.2–0.08)	0.01 (−0.62–0.64)	0.21	0.98
MF L_2_ contracted		2.76 (0.43)	2.81 (0.36)		3.34 (1.57)		2.80 (0.39)	−0.60 (−1.23–0.05)	0.03 (−0.60–0.66)	0.18	0.93
% change		5.90 (5.07)	6.14 (7.48)		5.86 (4.04)		4.30 (3.25)	0.01 (−0.62–0.64)	0.29 (−0.34–0.92)	0.98	0.27

MF L_3_ rest	28	2.93 (0.48)	2.93 (0.43)	15	2.98 (0.38)	15	2.97 (0.46)	−0.11 (−0.74–0.52)	−0.09 (−0.71–0.54)	0.72	0.79
MF L_3_ contracted		3.07 (0.49)	3.14 (0.42)		3.13 (0.38)		3.07 (0.44)	−0.14 (−0.77–0.49)	0.17 (−0.46–0.79)	0.65	0.62
% change		5.10 (4.11)	8.13 (8.03)		5.46 (3.64)		4.30 (4.60)	−0.09 (−0.72–0.54)	0.54 (−0.10–1.18)	0.77	0.05

MF L_4_ rest	28	3.40 (0.36)	3.41 (0.34)	15	3.46 (0.51)	15	3.48 (0.45)	−0.14 (−0.77–0.49)	−0.18 (−0.81–0.45)	0.70	0.60
MF L_4_ contracted		3.67 (0.36)	3.64 (0.37)		3.65 (0.51)		3.68 (0.47)	0.03 (−0.60–0.66)	−0.12 (−0.75–0.51)	0.93	0.73
% change		8.19 (7.30)	6.94 (5.52)		5.99 (4.97)		6.04 (3.36)	0.33 (−0.30–0.96)	0.18 (−0.45–0.81)	0.25	0.51

MF L_5_ rest	28	3.39 (0.41)	3.39 (0.43)	15	3.37 (0.33)	15	3.38 (0.46)	0.05 (−0.57–0.68)	0.03 (−0.60–0.66)	0.86	0.93
MF L_5_ contracted		3.68 (0.48)	3.66 (0.48)		3.65 (0.40)		3.62 (0.54)	0.08 (−0.55–0.70)	0.07 (−0.56–0.70)	0.81	0.83
% change		8.97 (6.44)	7.84 (4.65)		8.64 (6.71)		7.04 (5.30)	0.05 (−0.58–0.68)	0.16 (−0.47–0.79)	0.88	0.63

TrA rest	28	0.51 (0.09)	0.48 (0.08)	14	0.51 (0.14)	15	0.45 (0.10)	0.02 (−0.62–0.67)	0.36 (−0.27–0.99)	0.95	0.29
TrA contracted		0.70 (0.17)	0.71 (0.15)		0.72 (0.22)		0.66 (0.16)	−0.08 (−0.72–0.56)	0.33 (−0.31–0.96)	0.83	0.32
% change		38.23 (25.85)	49.19 (33.38)		41.35 (19.44)		46.50 (22.08)	−0.13 (−0.77–0.51)	0.09 (−0.54–0.72)	0.66	0.75

IO rest	28	1.20 (0.31)	1.29 (0.28)	14	1.07 (0.31)	15	1.18 (0.30)	0.39 (−0.26–1.04)	0.40 (−0.24–1.03)	0.24	0.23
IO contracted		1.36 (0.39)	1.48 (0.40)		1.23 (0.43)		1.36 (0.34)	0.33 (−0.32–0.98)	0.31 (−0.32–0.92)	0.34	0.31
% change		13.63 (9.19)	13.95 (11.55)		12.91 (11.90)		16.50 (12.56)	0.07 (−0.57–0.71)	−0.22 (−0.84–0.42)	0.85	0.52

Abbreviations: CI, confidence interval; IO, internal oblique; MF, multifidus; SD, standard deviation; TrA, transversus abdominis.

**TABLE 8:** Trunk muscle thickness and function of players who received a soft tissue injury in the playing season.

Variable	No soft tissue injury Mean (SD)			In‐season soft injury Mean (SD)				Effect size (CI 95%)		*p* value	
	*N*	Left	Right	*N*	Left	*N*	Right	Left	Right	Left	Right
MF L_2_ rest	35	2.69 (0.45)	2.71 (0.41)	8	3.37 (2.21)	8	2.61 (0.33)	−0.67 (−1.45–0.12)	0.25 (−0.52–1.02)	0.42	0.48
MF L_2_ contracted		2.83 (0.48)	2.83 (0.38)		3.51 (2.13)		2.73 (0.32)	−0.68 (−1.46–0.10)	0.28 (−0.49–1.05)	0.40	0.45
% change		5.82 (4.72)	5.74 (6.90)		6.20 (4.85)		4.47 (3.09)	−0.08 (−0.85–0.69)	0.20 (−0.57–0.97)	0.84	0.44

MF L_3_ rest	35	2.96 (0.45)	2.97 (0.42)	8	2.89 (0.43)	8	2.82 (0.50)	0.16 (−0.62–0.92)	0.34 (−0.43–1.11)	0.69	0.46
MF L_3_ contracted		3.11 (0.46)	3.15 (0.39)		3.03 (0.43)		2.96 (0.53)	0.18 (−0.60–0.95)	0.46 (−0.32–1.23)	0.65	0.36
% change		5.27 (3.79)	7.09 (7.60)		5.02 (4.68)		5.51 (5.38)	0.06 (−0.71–0.83)	0.22 (−0.55–0.99)	0.89	0.50

MF L_4_ rest	35	3.45 (0.38)	3.43 (0.35)	8	3.29 (0.56)	8	3.43 (0.51)	0.36 (−0.41–1.13)	0.02 (−0.75–0.79	0.49	0.97
MF L_4_ contracted		3.69 (0.38)	3.65 (0.51)		3.54 (0.56)		3.64 (0.51)	0.36 (−0.41–1.13)	0.03 (−0.74–0.80)	0.49	0.96
% change		7.36 (6.92)	6.65 (5.17)		7.73 (5.38)		6.54 (3.41)	−0.06 (−0.82–0.71)	0.02 (−0.75–0.79)	0.87	0.95

MF L_5_ rest	35	3.37 (0.40)	3.39 (0.45)	8	3.41 (0.29)	8	3.36 (0.41)	−0.10 (−0.86–0.67)	0.09 (−0.68–0.86)	0.77	0.82
MF L_5_ contracted		3.67 (0.47)	3.65 (0.51)		3.67 (0.47)		3.63 (0.43)	−0.01 (−0.78–0.76)	0.03 (−0.74–0.79)	0.97	0.94
% change		9.07 (6.78)	7.37 (4.84)		7.90 (5.06)		8.39 (5.10)	0.18 (−0.59–0.95)	−0.21 (−0.98–0.56)	0.59	0.62

TrA rest	35	0.51 (0.12)	0.48 (0.09)	7	0.50 (0.08)	8	0.46 (0.08)	0.05 (−0.77–0.86)	0.19 (−0.58–0.96)	0.89	0.60
TrA contracted		0.71 (0.20)	0.70 (0.16)		0.70 (0.12)		0.63 (0.11)	0.03 (−0.79–0.84)	0.46 (−0.32–1.23)	0.93	0.15
% change		39.08 (24.13)	50.43 (31.63)		40.20 (23.27)		38.73 (17.24)	−0.05 (−0.86–0.77)	0.39 (−0.38–1.17)	0.91	0.17

IO rest	35	1.17 (0.33)	1.27 (0.30)	7	1.11 (0.24)	8	1.18 (0.26)	0.17 (−0.64–0.99)	0.31 (−0.47–1.08)	0.61	0.41
IO contracted		1.31 (0.42)	1.46 (0.41)		1.33 (0.33)		1.36 (0.26)	−0.04 (−0.85–0.78)	0.26 (−0.51–1.03)	0.92	0.39
% change		12.21 (9.23)	14.25 (10.78)		19.30 (12.49)		17.42 (16.28)	−0.73 (−1.55–0.11)	−0.27 (−1.04–0.51)	0.20	0.61

Abbreviations: CI, confidence interval; IO, internal oblique; MF, multifidus; SD, standard deviation; TrA, transversus abdominis.

## 4. Discussion

This study provides the most comprehensive assessment of trunk muscle size and function and its relationship with lower limb injuries in AFL players to date. Results showed that a smaller CSA of the multifidus muscle and a larger CSA of the QL were associated with an increased risk of lower limb injury in male AFL athletes. A new finding was that players who sustained lower limb and soft tissue injuries had significantly smaller multifidus CSA at several vertebral levels. However, these findings should be interpreted with caution, as estimates were imprecise with wide confidence intervals that, in some cases, approached or crossed the null value. The magnitude of these odds ratios should also be interpreted cautiously, as effect sizes may be inflated in small samples with low event counts. The greatest between‐group differences in multifidus CSA were observed at the L_5_ lumbar vertebral level. Clinical cut‐off values were established, and values below the multifidus threshold and above the QL threshold were associated with higher odds of lower limb injury. While significant relationships were observed between multifidus and QL muscle CSA and injuries, there were limited significant associations between thickness measurements of the TrA, IO and multifidus muscles and playing season lower limb injuries.

### 4.1. Trunk Muscle Size and Lower Limb Injuries

The associations found between the CSA of the multifidus and QL muscle and lower limb injuries support previous research. In a study of 261 male AFL players, a similar relationship between a small multifidus CSA and in‐season lower limb injuries was found. Players with a multifidus muscle CSA below a clinical cut‐off of 8.5 cm^2^ were 12.2 times more likely to sustain a lower limb injury [13]. These results are similar to those of the current investigation, which observed a 10‐fold increase in lower limb injury risk among players below the 8.17 cm^2^ clinical cut‐off. In other sports, a relationship between multifidus muscle size and low back pain has been established [19–21], with a growing body of work finding a smaller multifidus CSA a risk factor for lower limb injury. In soccer players, findings have associated a decrease in multifidus CSA over the course of the season with lower‐limb injuries [22]. Rugby League players, using a higher cut‐off value (9.98 cm), showed a similar relationship between smaller multifidus muscles and injury [14]. The high odds ratio observed in the current and previous investigations supports further research into whether targeted interventions aimed at increasing multifidus muscle size could influence injury risk. The potential benefit of identifying clinical cut‐off values is that they may assist in identifying players who could be prioritised for further assessment and consideration of targeted interventions.

The current investigation provided a comprehensive assessment of the multifidus muscle and was able to compare CSAs across all lumbar levels. Significant odds ratios were found for the L_3_–L_5_ vertebral levels for lower limb injury risk and at L_5_ for soft tissue injury risk, with the most significant findings at the L_5_ vertebral level. This finding may be related to the anatomy of the multifidus muscle. The multifidus muscle is largest at the lumbosacral junction, with the erector spinae muscle group attaching to the erector spinae aponeurosis at the level of the L_4_ vertebrae [23]. Athletes are required to efficiently transfer force from the lower limbs across the lumbosacral junction to the upper limbs and vice versa. Suboptimal force transfer across the lumbopelvic region may contribute to an increased risk of injury. In support of this theory, reduced proprioception of the trunk was a predictive factor of knee injuries in collegiate athletes [9]. The reduced neuromuscular control of the trunk was theorised to have reduced the athlete’s capacity to accommodate large ground reaction forces directed towards the body’s centre of mass. This altered motor control may compromise the dynamic stability of the distal joints, including the knee joint. With the multifidus muscles having a large concentration of muscle spindles [24], their contribution to stabilising the lumbopelvic region is significant. This may explain why, particularly at the L_5_ vertebral level, the CSA of the lumbar multifidus is associated with lower limb injuries.

A larger QL was also significantly associated with lower limb injuries in the current investigation. Similar to our results, a previous study reported a clinical cut‐off value of 8.2 cm^2^, whereby players with a larger CSA of the QL muscle had a sevenfold increase in the odds of a preseason injury [13]. The explanation underpinning these findings is controversial, given the debate regarding the anatomy and function of the QL muscle. With the QL muscles exerting force in extension and lateral flexion [25], similar to the multifidus, a larger QL muscle could compensate for smaller multifidus muscles. Although the magnitude of force on the lumbar spine produced by the QL is less than 10% of the multifidus and erector spinae [26], a redistribution of force away from the multifidus and towards the QL may occur when the paraspinal muscles get fatigued. Fatigue can lead to altered neuromuscular control and altered spinal loading, leaving the spine and lower limbs susceptible to injury [26]. While it would be difficult to provide an intervention to reduce the size of muscles in athletes, monitoring changes in QL muscle size may provide further insight into the relationship between QL CSA and lower‐limb injuries in athletes.

### 4.2. Trunk Muscle Size and Clinical Cut‐Off Values

Clinical cut‐off values may provide a useful framework to assist in identifying players who could be prioritised for further assessment; however, these thresholds should be interpreted with caution. The cut‐off values identified in this study were derived from ROC analyses performed within the same cohort and are therefore subject to potential overfitting, which may increase the risk of type I error and reduce statistical power through dichotomisation of continuous variables. As such, these thresholds should be considered exploratory and hypothesis‐generating rather than definitive classification criteria. The primary findings of this study relate to the underlying continuous relationships between trunk muscle characteristics and injury risk, as reflected in the group comparisons, effect sizes and odds ratios. Validation of these cut‐off values in larger, independent cohorts is required before their use in clinical screening or decision‐making can be recommended. While these thresholds informed clinical decision‐making within the present applied setting, their use should remain cautious until validated in independent cohorts.

From a clinical perspective, these cut‐off values may assist in identifying players who warrant further assessment or targeted intervention within applied high‐performance environments. In the present study context, players identified as being at higher risk based on trunk muscle characteristics were prioritised for individualised physiotherapy input, including targeted exercise‐based interventions aimed at improving trunk muscle morphology and function. While the effectiveness of such interventions was not formally evaluated within this study, this approach illustrates a potential pathway from measurement to clinical decision‐making. As such, these findings may support the use of trunk muscle assessment as part of a broader screening and risk management strategy, with the aim of informing targeted injury prevention interventions.

The highest AUC values for multifidus muscle CSA were observed at the L_5_ vertebral level for lower‐limb injuries and at L_2_ and L_5_ for soft tissue injuries. The AUC for QL muscle CSA was 0.78 with a clinical cut‐off value at 8.44 cm^2^. Players above this cut‐off had a sevenfold increase in lower limb injury risk across the season, supporting a previous result of a cut‐off of 8.2 cm^2^ with a sevenfold increase in injury risk [13]. Both multifidus and QL muscle cut‐off values were found to be significant in a professional male AFL team; however, the results require validation in independent AFL cohorts before their use as screening thresholds can be considered.

### 4.3. Trunk Muscle Function and Lower Limb Injury

Measurements of contraction of the IO, TrA and multifidus muscles were not associated with sustaining a lower‐limb or soft tissue injury in the AFL players studied. There was no indication that the ability to contract the IO, TrA or multifidus muscles was protective or a risk factor for injury. Previous investigations of muscle function and lower limb injury have found associations with patellofemoral pain [18], knee pain in badminton players [16] and poorer performance on the landing error scoring system [17]. Studies using electromyography have also demonstrated associations between trunk activation patterns and lower limb injury [27, 28]. While the current findings do not support these associations, differences may be explained by the methods used to assess muscle function. Measures of voluntary isometric contraction, such as those used in the present study, are dependent on participant understanding, effort and consistency, which may introduce variability. In contrast, techniques such as electromyography may provide a more sensitive measure of neuromuscular activation. These findings suggest that trunk muscle morphology and function may represent different aspects of neuromuscular performance and that measures of muscle size, which are thought to be related to strength [29], may provide a more robust and reproducible marker in applied screening settings. Further research is required to clarify the role of trunk muscle function in injury risk and to determine the most appropriate methods for its assessment. These findings should be interpreted with caution, as the use of unadjusted analyses may be influenced by unmeasured confounding factors, including workload, previous injury and playing position.

### 4.4. Limitations

This study was conducted within a single professional AFL team, which may limit the generalisability of the findings to other teams, competitions, or athlete populations. Player exposure to training and matches was not accounted for, limiting the ability to contextualise injury risk relative to workload. Without exposure data, injury risk could not be expressed relative to time at risk, which may limit comparability and introduce bias. Additionally, previous injury history was not included in the analysis, despite being a known risk factor for future injury. Positional demands in AFL may influence both trunk muscle characteristics and injury risk; however, player position was not analysed in this study and represents a potential source of variability for future research.

The relatively small sample size and limited number of injury events, particularly for soft tissue injuries (*n* = 8), reduced the statistical power of subgroup analyses and increased uncertainty around the effect estimates, as reflected in the wide confidence intervals. This also restricted the ability to perform multivariable modelling to adjust for potential confounders, as the number of events was insufficient to support stable estimates. Consequently, unadjusted analyses were presented, which may increase susceptibility to both type I and type II errors. Future studies with larger cohorts are required to allow appropriate adjustment for confounding variables. Although injury mechanism (e.g., contact vs. noncontact) was not explicitly analysed, the soft tissue injury subgroup likely reflects predominantly noncontact mechanisms; however, further subdivision was not feasible due to the small number of injury events.

Injury classification was based on match time‐loss and did not include detailed information regarding injury severity, mechanism, or recurrence. While this reflects a pragmatic approach commonly used in applied sporting environments, it may oversimplify the complexity of injury outcomes and limit the ability to examine more specific injury patterns. In addition, contact and noncontact injuries were not analysed separately, which may reduce specificity when interpreting intrinsic risk factors. Due to the small number of injury events, further stratification was not feasible. Future studies should incorporate more detailed injury classification and exposure‐based metrics to better characterise injury risk.

Measurements in this study were obtained at a single time point during the preseason and do not account for changes in muscle morphology and function across the playing season. Given that both trunk muscle characteristics and injury risk are dynamic and multifactorial, this limits the ability to capture temporal relationships and precludes causal inference. Accordingly, the findings should be interpreted as associative rather than causal. Future research incorporating longitudinal designs with repeated measurements is needed to better understand how changes in trunk muscle characteristics relate to injury risk over time.

## 5. Conclusion

This study demonstrates that trunk muscle morphology is associated with the risk of lower‐limb injury in elite AFL players and provides preliminary, clinically interpretable cut‐off values that require validation. Although these thresholds were derived within a single cohort and require validation, they offer a practical foundation for use in applied settings.

The findings also suggest a clear pathway for clinical translation, whereby trunk muscle assessments can be used to guide targeted physiotherapy interventions for players identified as higher risk. While the effectiveness of such interventions was not evaluated in this study, these results support the integration of trunk muscle assessment into screening and injury prevention strategies within elite sport.

## Author Contributions

All authors contributed to the study conception and design. Material preparation, data collection and analysis were performed by Clint Frazer, M. Dilani Mendis and Julie A. Hides. The first draft of the manuscript was written by Clint Frazer, and all authors commented on previous versions of the manuscript.

## Funding

This project was supported by Griffith University Health Group & Brisbane Lions FC: Fostering Research Partnerships Scheme.

Open access publishing is facilitated by Griffith University, as part of the Wiley–Griffith University agreement via the Council of Australasian University Librarians.

## Disclosure

All authors read and approved the final manuscript.

## Ethics Statement

This project was supported by Griffith University Health Group & Brisbane Lions FC: Fostering Research Partnerships Scheme. Informed consent was obtained from all individual participants involved in the study. All procedures performed in this study were in accordance with the ethical standards of the institutional and/or national research committee.

## Consent

The authors affirm that human research participants provided informed consent for publication of all images and data within this article.

## Conflicts of Interest

The authors declare no conflicts of interest.

## Supporting Information

Additional supporting information can be found online in the Supporting Information section.

## Supporting information


**Supporting Information** STROBE_Checklist_Cohort_3.

## Data Availability

The data that support the findings of this study are available from the corresponding author upon reasonable request.
