# Supplementary material for: Developing Clinically Relevant Cut‐Off Values for Trunk Muscle Size in Relation to Lower Limb Injuries in AFL Players
Source: Transl Sports Med. 2026 Sep 21;2026:5549825. doi: 10.1155/tsm2/5549825 (PMC13594380; doi:10.1155/tsm2/5549825)
Supplement: Supplementary file 1 [file TSM2-2026-5549825-s001.docx]

STROBE Checklist – Cohort Study

| Item | Recommendation | Reported (Yes/No/Partial) | Page/Section/Notes |
| --- | --- | --- | --- |
| 1a | Study design in title/abstract | Yes | Abstract |
| 1b | Informative abstract | Yes | Abstract |
| 2 | Background/rationale | Yes | Introduction |
| 3 | Objectives/hypotheses | Yes | Introduction |
| 4 | Study design early in paper | Yes | Abstract/Methods |
| 5 | Setting, location, dates | Yes | Methods (team/season described; dates provided) |
| 6a | Eligibility criteria | Yes | Methods – Participants |
| 6b | Matching criteria | N/A | Not matched |
| 7 | Variables defined | Yes | Methods |
| 8 | Data sources/measurement | Yes | Methods |
| 9 | Bias addressed | Yes | Limitations |
| 10 | Study size | Yes | Statistical Analysis (sample size justification provided) |
| 11 | Quantitative variables | Yes | Statistical Analysis |
| 12a | Statistical methods | Yes | Statistical Analysis |
| 12b | Subgroups/interactions | Yes | Results |
| 12c | Missing data | Yes | Statistical Analysis |
| 12d | Loss to follow-up | N/A | Methods |
| 12e | Sensitivity analyses | Partial | Exploratory subgroup analyses (soft tissue injuries) |
| 13a | Participant flow | Yes | Flow chart included |
| 13b | Non-participation reasons | Yes | Methods (no players declined participation) |
| 13c | Flow diagram | Yes | Methods, Section 2.1 Participants10 |
| 14a | Participant characteristics | Yes | Results |
| 14b | Missing data per variable | Yes | Results and Statistical analysis |
| 14c | Follow-up time | Yes | Results |
| 15 | Outcome data | Yes | Results |
| 16a | Main results (estimates + CI) | Yes | Results |
| 16b | Category boundaries | Yes | ROC analysis |
| 16c | Absolute risk | Partial | Relative risk only |
| 17 | Other analyses | Yes | Results |
| 18 | Key results summary | Yes | Discussion |
| 19 | Limitations | Yes | Discussion |
| 20 | Interpretation | Yes | Discussion |
| 21 | Generalisability | Yes | Discussion |
| 22 | Funding | Yes | Declarations |
